# A longitudinal study of burden among spouse and non-spouse caregivers of older adults with stroke-induced-dependency

**DOI:** 10.1590/0034-7167-2023-0052

**Published:** 2023-12-04

**Authors:** Carolina Baltar Day, Carla Cristiane Becker Kottwitz Bierhals, Fernanda Laís Fengler Dal Pizzol, Gail Low, Naiana Oliveira dos Santos, Lisiane Manganelli Girardi Paskulin

**Affiliations:** I Pontifícia Universidade Católica do Rio Grande do Sul. Porto Alegre, Rio Grande do Sul, Brazil; II Universidade Federal do Rio Grande do Sul. Porto Alegre, Rio Grande do Sul, Brazil; III University of Alberta. Edmonton, Alberta, Canada; IV Universidade Franciscana. Santa Maria, Rio Grande do Sul, Brazil; V Hospital de Clínicas de Porto Alegre. Porto Alegre, Rio Grande do Sul, Brazil

**Keywords:** Aged, Caregiver Burden, Caregivers, Nursing, Stroke, Pessoa Idosa, Fardo do Cuidador, Cuidador Familiar, Enfermagem, Acidente Vascular Cerebral, Anciano, Carga del Cuidador, Cuidador Familiar, Enfermería, Accidente Cerebrovascular

## Abstract

**Objective:**

to assess the burden of spouse and non-spouse caregivers of older adults with stroke-induced-dependency after discharge from a university hospital’s Specialized Care Stroke Unit in southern Brazil.

**Methods:**

a longitudinal survey. The sample consisted of 48 consenting caregivers, among which 20 were spouse caregivers. Data were collected between May 2016 and July 2018. One week after discharge, caregivers completed a sociodemographic profile, the Functional Independence Measure, and the Caregiver Burden Scale. Burden was also measured two months after discharge. Data were analyzed using Multivariate Analyses of Variance.

**Results:**

regarding time 1, non-spouse caregivers experienced greater burden with respect to social isolation (p = .01). Along with a persistently greater sense of isolation (p=.04), non-spouse caregivers felt far greater general strain (p =.01).

**Conclusion:**

statistically significant differences in burden over time highlight the importance of assessing caregiver burden after discharge and the need for a formal support program.

## RESUMO

**Objetivo:**

avaliar sobrecarga de cuidadores cônjuges e não cônjuges de idosos dependentes por AVC pós-alta da Unidade de Atendimento Especializado em AVC de um hospital do sul do Brasil.

**Método:**

pesquisa longitudinal. Amostra foi composta por 48 cuidadores, sendo 20 cuidadores cônjuges. Dados foram coletados entre maio/2016 e julho/2018. Uma semana pós-alta, verificou-se a capacidade funcional dos idosos pela Medida de Independência Funcional, o perfil sociodemográfico dos cuidadores e a sobrecarga com a Caregiver Burden Scale. A sobrecarga também foi medida dois meses pós-alta. Dados foram analisados por meio de Análise Multivariada de Variância.

**Resultados:**

no tempo 1, não cônjuges apresentaram maior sobrecarga em relação ao isolamento social (p=0,01). Juntamente com isolamento (p=0,04), não cônjuges sentiram tensão geral muito maior (p=0,01).

**Conclusão:**

diferenças estatisticamente significativas na sobrecarga ao longo do tempo destacam a importância de avaliar a sobrecarga do cuidador pós-alta e a necessidade de um programa formal de apoio.

## RESUMEN

**Objetivo:**

evaluar la carga de los cuidadores conyugales y no conyugales de ancianos con dependencia inducida por ictus después del alta de la Unidad de Atención Especializada en Ictus de un hospital del sur de Brasil.

**Métodos:**

una encuesta longitudinal, con 48 cuidadores (20 cónyuges). Datos fueron recolectados entre mayo/2016 y julio/2018. Una semana después del alta, se aplicó la Medida de Independencia Funcional a los ancianos y la Caregiver Burden Scale a los cuidadores. Los datos se analizaron mediante análisis multivariado de varianza.

**Resultados:**

en tiempo 1, los cónyuges experimentaron mayor carga en relación al aislamiento social (p=0,01). Los cónyuges sintieron una tensión general y sensación de aislamiento mucho mayor (p=0,01; p=0,04).

**Conclusión:**

las diferencias estadísticamente significativas en la carga a lo largo del tiempo resaltan la importancia de evaluar la carga del cuidador después del alta y la necesidad de un programa de apoyo formal.

## INTRODUCTION

Stroke is a primary cause of physical disabilities, especially in older people^(1)^. Stroke survivors present severe physical, cognitive and behavioral sequels that negatively affect the performance of activities of daily living, such as bathing, eating, and dressing^(2)^. Transition of care in Brazil is still incipient to support programs and health services for older adults with stroke-induced-dependency transitioning from hospital to home^(3)^.

Caring for older adults with stroke-induced-dependency is solely a family matter^(4)^. Some family caregivers report a lack of knowledge related to the bodily impacts of stroke and appropriate restorative care at home^(5-6)^. Historically, women are more likely to be informal caregivers in Latin American culture^(7)^. There is a hierarchy of commitment in relation to care, being the responsibility of wives first, followed by unmarried daughters^(8)^. Currently, studies show that spouses represent a significant portion of caregivers^(3,9)^.

In this context, caring for an older person after a stroke involves carrying out daily tasks related to activities of daily living such as hygiene, food, mobilization and locomotion. The provision of care to older adults left with significant physical and cognitive impairments is a strong predictor of perceived burden^(10)^. A Canadian study identified that spouses who care for dependent older people have greater social isolation and financial difficulties than non-spouse caregivers^(11)^. Italian research showed that spouses of patients with moderate to severe brain injury had a high level of burden. The more severe the injury, the greater the negative impact on the family routine and the greater burden on the spouse^(12)^. In the Brazilian scenario, a study with informal caregivers of older people identified a greater burden on spouses. For them, the act of caring involved their personal life, resulting in a high physical, emotional and social burden^(13)^.

The burden faced by family caregivers of older stroke survivors is well documented in the literature, especially with regard to the moment of transition from specialized care to that to be performed at home^(14)^. However, studies related to burden assessment in spouse and non-spouse caregivers and how it changes over time are lacking. Such studies are needed to better understand how caregiver burden affects the daily lives of family members taking over a caregiver role for older adults with stroke-induced-dependency. The findings of this study could help nurses better anticipate burden-related needs among such caregivers.

## OBJECTIVE

To assess perceived burden among spouse and non-spouse caregivers of older adults with stroke-induced-dependency after discharge from a Specialized Stroke Care Unit.

## METHODS

### Ethical aspects

The study was approved by the Research Ethics Committee (redacted). All family caregivers provided informed consent before completing any study questionnaires.

### Study design, period and place

This study makes use of longitudinal survey data collected in an earlier family caregivers’ quality of life study^(15)^, and was structured with the STrengthening the Reporting of OBservational studies in Epidemiology (STROBE) recommendations^(16)^. Caregivers taking part in this parent study had a loved one who was being cared for in a Specialized Care Stroke Unit (SCU-Stroke) at a university hospital in southern Brazil. Parent study data were collected between May 2016 and July 2018 by trained undergraduate research assistants.

### Population and sample; inclusion and exclusion criteria

Eligibility criteria for family caregivers in the parent study were: 18+ years of age; taking care of older adults who had experienced a stroke for the first time and who remain with significant stroke-related functional impairments. Level of independence among older adults with stroke-induced-dependency was assessed using the Brazilian version of the Functional Independence Measure (FIM). The FIM measures self-care, sphincter control, mobility, locomotion, communication, and social cognition^(17)^. Each item is measured on a Likert-type scale ranging from 1 to 7 (1: Total Dependency; 2: Maximal Dependency; 3: Moderate Assistance; 4: Minimal Assistance; 5: Supervision; 6: Modified Independence; and 7: Complete Independence). Scores across all six dimensions are added to generate a total FIM score and can range from 18 to 126^(17-18)^. Exclusion criteria were: stroke survivor living in a long-term care facility; or being followed by Home Care Services after discharge.

At the time the parent study took place, a total of 471 patients were admitted to the SCU-Stroke. Ninety-two of the 245 patients admitted due to having had a stroke were eligible to take part. Excluded patients were decedents (n=8) or long-term care residents (n=15), or Home Care Service candidates (n=8). Thirteen family caregivers did not wish to participate. Among the 48 family caregivers consenting to take part in the parent study, 20 were spouses. Spouse and non-spouse caregivers were followed up approximately one week (Time 1) and two months (Time 2) after discharge.

### Study protocol

Family caregivers were identified at the time of discharge from SCU-Stroke. Participants who met the eligibility criteria and agreed to participate in the study signed the Informed Consent form. Within 7 days (Time 1) of hospital discharge, a home visit to family caregiver was performed to collect their sociodemographic and clinical data and verified caregiver burden. In two months (Time 2), a second home visit was performed to assess caregiver burden. The home visit was previously scheduled by telephone contact at a date and time comfortable for caregivers.

For sociodemographic and clinical characterization, caregivers were asked about their biological sex, age, education, family income, numbers of chronic illnesses, and caregiving experiences. We were also particularly interested in whether caregivers had previous caregiving experience, how many days and hours per day they were providing care, and whether they lived with an older adult with stroke-induced-dependency. Family caregivers also indicated the type of help they were receiving from people within their own support networks. Family support can involve giving and receiving financial help, instrumental help related to the provision of direct care, and emotional help^(3)^.

Spouse and non-spouse caregiver burden was measured using the 22-item Brazilian Caregiver Burden Scale (CBS)^(19)^. Caregivers were asked about their health, mental well-being, personal relationships, physical burden, social support, finances, and environment. Items refer to self-reported General Strain, Isolation, Disappointment, Emotional Involvement, and Environment. All items are measured on a 4-point Likert scale (1 = not at all; 2 = seldom; 3 = sometimes; 4 = often). Items within each domain are added to yield a total CBS score, with a higher total score indicating higher perceived burden^(19)^.

Medeiros *et al*. (1998) provided evidence of inter-rater reliability (range = 0.87 to 0.92) of the CBS among caregivers of older people with rheumatoid arthritis. In Brazil, other studies also used the CBS validated by Medeiros *et al*. (1998)^(19)^. Cavalari and *et al*. (2017) report a total score reliability coefficient (Cronbach’s alpha = 0.88) among informal caregivers of children after postnatal spinal dysraphism correction. In their study of caregivers of individuals with spinal cord injury^(20)^, Nogueira *et al*. (2013) reported Cronbach’s alpha coefficients ranging from 0.35 to 0.88. To the best of our knowledge, internal reliability coefficients for the CBS have not yet been reported among Brazilian informal caregivers of older adults after a stroke^(21)^.

Internal consistency reliability coefficients for the non-Brazilian CBS have ranged from 0.70 to 0.87 among informal caregivers of older adults with stroke-induced-dependency^(22)^. In Farajzadeh *et al*. (2018) study of Iranian caregivers of patients with spinal cord injury, the environmental domain alone exhibited low internal consistency reliability (alpha = 0.559)^(23)^. In a Swedish randomized control trial for family caregivers in palliative home care^(24)^, Cronbach’s alpha coefficients ranged from 0.70 (Environment) to 0.87 (General Strain).

Although the Brazilian CBS^(19)^ has not yet been validated among informal caregivers of older adults with stroke-induced-dependency, this instrument was chosen as it was found to be a reliable instrument in the original study^(22)^ and in others’ studies^(20-21,23-24)^. Moreover, this scale has been widely used in other national and international studies of caregivers of older adults with stroke-induced-dependency^(25-26)^.

In other caregiver burden scales that have been validated for use in Brazil, there is no environmental domain^(27-29)^. The CBS assesses environmental issues related to: (a) difficulties encountered in the physical environment to provide care (presence of stairs, size of the physical space for patient mobility, bathroom without adaptations to assist in patient hygiene, etc.); (b) structure provision of services in the neighborhood where they live (difficulty in using public transport, difficult access to pharmacies and/or health care services, problems with neighbors, noise, sanitary conditions, etc.); and (c) caregivers’ cognitive-affective perception in relation to the how they are offering care to their family member.

In this study, Cronbach’s alpha coefficient for General Strain, Isolation, Disappointment, Emotional Involvement, and Environment domains at Time 1 were 0.887, 0.560, 0.680, 0.654, 0.530, respectively. At Time 2, these coefficients were 0.893, 0.449, 0.736, 0.819, and 0.158, respectively. In keeping with these mixed findings, our discussion of the findings concerns the General Strain domain, as this domain showed acceptable internal consistency (Cronbach’s alpha > 0.70)^(30)^ at Time 1 and 2. Furthermore, we opted to discuss the Isolation domain because this domain yielded the most statistically significant findings.

### Analysis of results, and statistics

Study data were analyzed using the Statistical Package for the Social Sciences version 22.0 software. Spouse and non-spouse characteristics, caregiving experiences, burden, and functional capacity of older adults with stroke-induced-dependency were quantified using categorical frequencies and descriptive statistics. Independent Student’s t-tests were used to compare spouse versus non-spouse caregivers’ age, family income, level of education, days and hours caring for the older adults with stroke-induced-dependency, numbers of chronic illnesses, perceived burden, and functioning levels among older adults with stroke-induced-dependency. Chi-square or Fisher’s exact test were used for comparisons based on biological sex, living with older adults with stroke-induced-dependency (yes/no), previous experience in caring (yes/no), and types of informal support (instrumental, emotional, and financial).

Multivariate Analyses of Variance (MANOVA) allowed comparisons of perceived burden among spouse versus non-spouse caregivers. Remarkably different caregiver characteristics were treated as covariates in global burden and item-specific burden MANOVA.

## RESULTS

Family caregiver characteristics are presented in Table 1. Caregivers were predominantly female across both groups (p=0.68). Spouse caregivers were significantly older (p<.001) and significantly less educated (p=.002) than non-spouse caregivers. Every spouse caregiver was living with an older adult with stroke-induced-dependency. Nearly all spouse caregivers had previous caregiving experience (p<.001).

**Table 1:** Family Caregiver Characteristics

Variables	Time^†^	Spouse (n=20) n(%)	Non-spouse (n=28) n(%)	*p* value
Sex				
Female	Time 1	17(85)	25(89.3)	0.683
Male	Time 1	3(15)	3(10.7)	
Living with the older adult with stroke-induced-dependency				
Yes	Time 1	20(100)	23(82.1)	0.066
Experience in caring				
Yes	Time 1	19(95)	12(42.9)	0.000^^*^^
Kind of help received				
Instrumental	Time 1	15(75)	24(85.7)	0.460
	Time 2	19(95)	22(91.7)	1.000
Emotional	Time 1	13(65)	20(71.4)	0.636
	Time 2	16(80)	13(54.2)	0.072
Financial	Time 1	4(20)	16(57.1)	0.010^^**^^
	Time 2	5(25)	15(65.2)	0.013^^***^^

† Time 1: one week after SCU-Stroke discharge. Time 2: two months after SCU-Stroke discharge.

* p<.001;

** p<.01;

*** p<.05

At Time 1 and 2, there were no statistically significant differences between caregiver groups in terms of having versus not having instrumental and emotional help. Non-spouses were more likely to have financial help at Time 1 (57.1% vs 20%, p=.010) and at Time 2 (65.2% vs 25%, p=.013). There were no statistically significant differences between caregiver groups with respect to monthly family income (p=.105) and hours of caring (p=.756). At Time 1, older adults with stroke-induced-dependency who were being cared for by non-spouse versus spouse caregivers had significantly lower FIM scores (p=.013).

### Domain-specific MANOVA

CBS scores for spouse and non-spouse family caregivers are presented in Table 3. These MANOVA derived mean scores were adjusted for the remarkably different caregiver characteristics and the functional capacities of older adults with stroke-induced-dependency shown in Table 2. Non-spouse caregivers had statistically significantly higher Isolation scores at Time 1 and Time 2, as was the case for General Strain at Time 2.

**Table 2:** Family Caregiver Characteristics and Experiences, and Functional Capacity of Older Adults with Stroke-Induced-Dependency

Variables	Spouse (n=20) mean (SD)	Non-spouse (n=28) mean (SD)	Mean difference (95% CI)	*p* value
Caregivers				
Age^†^	61.30 (10.08)	47.86 (11.80)	13.44 (6.88 to 20.00)	0.000^^*^^
Family income^‡^	2487.30 (1256.28)	1.951 (818.66)	535.87 (-118.80 to 1190.54)	0.105
Education^§^	6.28 (4.08)	10 (3.86)	-3.76 (-6.09 to -1.42)	0.002^^**^^
Hours of caring^†^	19.00 (6.50)	19.57 (6.03)	-0.57 (-4.24 to 3.10)	0.756
Older adults with stroke-induced-dependency				
Functional Capacity	67.25 (19.98)^†^	52.64 (18.80)^†^	-14.60 (-25.98 to -3.23)	0.013^^***^^
	82.90 (24.10) ^¶^	68.82 (25.90)^¶^	-14.07 (-29.92 to -0.76)	0.063

SD: Standard Deviation. CI: Confidence Interval;

† Time 1: one week after SCU discharge;

* p<.001; ‡ Family monthly income based on the National Brazilian minimum wage in 2018 (R$937.00);

§ Education in years;

** p<.01;

*** p<.05;

¶ Time 2: two months after SCU discharge

**Table 3:** CBS Scores for Spouse and Non-Spouse Family Caregivers

Domain	Spouse (n=20) mean (SD)	Non-spouse (n=28) mean (SD)	Mean differences 95% (CI) ^†^	*p* value^‡^
Isolation	4.61±0.52^§^	6.27±0.49^§^	1.65 (-0.02 to 3.28)	0.046^^*^^
	4.70±0.52^¶^	6.69± 0.57^¶^	1.96 (0.11 to 3.80)	0.038^^*^^
General Strain	14.20±1.45^§^	16.81±1.38^§^	2.62 (-1.90 to 7.13)	0.249
	13.24±1.53^¶^	20.47±1.69^¶^	7.23 (1.82 to 12.64)	0.010^^*^^
Disappointment	8.52±0.73^§^	9.36±0.69^§^	- 0.84 (-1.14 to 3.12)	0.462
	8.09±0.86^¶^	11.09±0.95^¶^	2.99 (0.71 to 6.06)	0.055
Emotional involvement	3.55±0.30^§^	3.42±0.288^§^	0.10 (-0.84 to 1.04)	0.830
	3.23±0.41^¶^	3.92±0.45^¶^	0.68 (0.76 to 2.14)	0.345
Environment	5.02±0.55^§^	5.90±0.52^§^	-0.87 (-2.60 to 0.84)	0.310
	4.57±0.42^¶^	5.53±0.46^¶^	0.95 (2.46 to 0.59)	0.206

SD: Standard Deviation. CI: Confidence Interval;

† 95% confidence interval. CBS scores were adjusted for functional capacity of older adults with stroke-induced-dependency and caregiver age, education, living arrangements, financial support, and previous experience;

‡ MANOVA or Multivariate Analyses of Variance;

§ Time 1: one week after SCU discharge;

* p<0.05;

¶ Time 2: two months after SCU discharge.

### Item-specific MANOVA

To shed further light on these differences between groups, we examined item-specific patterns of scores for Isolation and General Strain (Table 4). Within the Isolation domain, non-spouses had higher scores for item 10 (Has your social life - with family and friends - been lessened?) at Time 1 and item 11 (Has your relative’s problem prevented you from doing what you had planned to do in this phase of your life?) at Time 2. This group also had the highest General Strain scores for items 1 (Do you find yourself facing purely practical problems in the care of your relative that you think are difficult to solve?), 5 (Do you feel tied down by your relative’s problem?), 7 (Do you think your own health has suffered because you have been taking care of your relative?), and 8 (Do you think you spend so much time with your relative that the time for yourself is insufficient?) at Time 2.

**Table 4:** CBS Item Scores for Spouse and Non-Spouse Family Caregivers

Domain/ Item	Item question			*p* value^‡^
	Spouse (n=20) mean (SD)	Non-spouse (n=28) mean (SD)	Mean differences 95% (CI) ^†^	
Isolation				
Item 1^0^§ Has your social life (with family and friends) been lessened?	1.69±0.28	2.76±0.26	1.07 (0.19 to 1.95)	0.017^^*^^
Item 1^1^¶ Has your relative’s problem prevented you from doing what you had planned to do in this phase of your life?	1.78±0.26	2.99±0.29	1.20 (0.27 to 2.13)	0.013^^*^^
General strain				
Item 1^¶^ Do you find yourself facing purely practical problems in the care of your relative that you think are difficult to solve?	1.40±0.25	2.93±0.28	1.53(0.61 to 2.44)	<0.01^^**^^
Item 5^¶^ Do you feel tied down by your relative’s problem?	1.52±0.25	2.60±0.28	1.08 (0.18 to 1.98)	0.020^^*^^
Item 7^¶^ Do you think your own health has suffered because you have been taking care of your relative?	1.27±0.21	1.98±0.23	0.76 (0.005 to 1.51)	0.049^^*^^
Item 8^¶^ Do you think you spend so much time with your relative that the time for yourself is insufficient?	1.30±0.25	2.44±0.28	1.13 (0.22 to 2.05)	0.016^^*^^

SD: Standard Deviation. CI: Confidence Interval;

† 95% confidence interval. CBS scores were adjusted for functional capacity of older adults with stroke-induced-dependency and caregiver age, education, living arrangements, financial support, and previous experience;

‡ MANOVA or Multivariate Analyses of Variance;

§ Time 1: one week after SCU discharge; ^^*^^ p<0.05;

¶ Time 2: two months after SCU discharge; ^^**^^ p <0.01.

## DISCUSSION

The aim of this Brazilian study was to shed light on perceived burden among spouses and non-spouses who take over caregiver roles for older adults with stroke-induced-dependency.

### Time 1 Isolation

In Brazil, providing full-time care for an older adult with stroke-induced-dependency is a reality for many families, with the lack of formal care support and cultural expectations being primary determining factors. Stroke is associated with a number of physical and psychological problems that understandably restrict caregivers’ social lives. For example, the dependence of the older adult to develop basic and instrumental activities of daily living, require caregivers to perform care that includes bathing, changing diapers, handling bladder and feeding tubes, mobilizing in bed, transferring from bed to chair, taking care with medication administration, preventing pressure injuries, among others, all of which imply a need for constant caregiver assistance^(31-32)^. Moreover, in the home care for an older adult with stroke-induced-dependency can be identified factors of burden to caregiver, such as the lack of information and skills for care, fragility in the support, and social network^(33)^.

Caregiver work can be lonely work as caregiving work is typically undertaken by a single family member^(34)^. At Time 1, one-week after discharge, non-spouse caregivers were already experiencing far greater isolation, partly with respect to limited or lost social connections with friends and other family. Older adults who were being cared for by a non-spouse exhibited significantly lower levels of functional impairment. Spouse caregivers’ social limitations might have been mitigated through affectionate exchanges with their ill partner. Marital quality can influence spouses’ loneliness, with dissatisfied spouses tending to experience greater loneliness^(35)^. Non-spouses may therefore have had greater difficulty adjusting to being disconnected from broader social circles, such as co-worker friends, or their own spouse or children. We suspect that older spouses’ social activities primarily revolve around one another.

### Time 2 Isolation

At time 2, two months after discharge, higher perceived isolation among non-spouse caregivers persisted, with this now pertaining to not being able to do what they wanted to do at their current phase of life. In a qualitative study of older Nigerians with chronic illnesses, family caregivers expressed a deep sense of abandonment and isolation^(36)^. Family members who should have been supporting caregivers physically, financially or emotionally were simply not there. Adult child and spouse caregivers were expected to balance care recipients’ needs with their own needs and wants, and to do so aptly while all alone. All non-spouse caregivers were more inclined than spouse caregivers to be receiving instrumental assistance one week and two months after taking over an in-home caregiver role, and slightly less emotional support at month two.

In Brazilian society, it is considered socially inappropriate and embarrassing to admit that their spouses prevent them from pursuing other supposedly more palatable social activities. Caregiving is part-and-parcel of tending to their own household. Spouses who have alternate caregivers are more likely to be held in ill rather than in high regard. Concurrent developmental stressors such as job losses, financial constraints, and mentoring and caring for younger family members are typical in the 4^th^ and 5^th^ decades of life^(37)^. Non-spouse caregivers may have been experiencing concurrent difficulties such as postponement or modification of professional goals.

In Brazil, there are no financial support programs for workers who take over full-time caregiver roles, nor are there practical support such as flexible work hours. Perhaps this is why non-spouse caregivers received significantly more financial support than spouse caregivers. Losses in social status in one’s 40s can have lingering detrimental effects on positive and negative effects^(38)^ and worthwhileness in life^(39)^ in one’s 50s. How non-spouse caregivers negotiate paid worker and unpaid caregiver roles bodes empirical attention. Midlife adults often feel that their time and energy is pulled in competing directions at home and at work^(40)^. To our knowledge, no Brazilian studies have been undertaken among typically younger non-spouse caregivers.

### Time 2 General Strain

Although caregiving for an older adult with no prior strokes could be construed a new undertaking, in Brazilian society, spouses provide the lion’s share of caregiving within the family unit. Caregivers of older adults with stroke-induced-dependency who have versus do not have caregiving experience seem better able to identify coping strategies and see caregiving in a more positive light^(41)^. In this study, spouse caregivers were significantly older and had more prior experience. Older caregivers who identify with cultural traditions also tend to be more positive about caregiving than their younger counterparts^(41)^. Perhaps this is why spouse caregivers experienced lesser General Strain.

Non-spouse caregivers faced strains of greater significance to them alone. Non-spouse caregivers experienced greater difficulties with practical problems arising from care, lack of time for themselves, and own-health deterioration. Midlife adults can feel torn between protecting others’ health and well-being and avoiding self-neglect^(42)^. In a Taiwanese study of older adults with stroke-induced-dependency, first-time family caregivers who tended to define their own physical or mental health as poor also expressed greater burden^(43)^. Older adults with stroke-induced-dependency being cared for by non-spouses did show far lower functional capacities one week after discharge. Seven weeks later, the similar improvements in stroke survivor functioning across both caregiver groups did not ameliorate non-spouse caregivers’ far greater sense of burden. As first-time caregivers, non-spouses also expressed a significantly greater sense of feeling ‘tied down’ by a relative’s health problem. Along with a lingering sense of isolation from family, friends and plans for their own lives, non-spouse caregivers were privy to multiple simultaneous sources of strain.

### Study limitations

The study has limitations that prevent us from generalizing our findings beyond the studied sample. Family caregivers were recruited from a SCU-Stroke unit and all such caregivers also live in South Brazil, a part of the country wherein people tend to be older. A larger sample from multiple and non-specialized health services and geographic regions is needed.

Our findings are also limited by the CBS’ less than ideal internal consistency reliability. In Brazil, female caregivers in particular are expected to muffle their emotions, make do with having a lack of time for themselves, and abandon their personal or professional endeavors^(44)^. Women are also expected to express gratitude for being a caregiver because doing so makes you a “good person”^(44)^. We therefore suspect that the predominantly female caregivers in this study may have held back their true feelings about isolation. The low Cronbach’s alpha coefficient for isolation in this study may therefore be a cultural artifact. The Isolation domain also contains three items whereas the General Strain domain has eight items. Three items may be inadequate to comprehensively assess isolation among caregivers^(45)^.

Further studies will help nurse researchers to better understand the internal consistency of the CBS within a Brazilian context. Qualitative studies of the meaning of isolation among non-spouse caregivers could generate additional items that resonate with this typically younger caregiver population. How non-spouse caregivers make ends meet could be especially informative. Non-spouse caregivers in this study were more likely to be receiving financial support from others. Unfortunately, the CBS does not include questions about work or finances.

In keeping with our said limitations, our findings should be interpreted with caution. Nonetheless, we hope that this novel longitudinal study enhances nurse researchers’ interests in measuring and assessing caregiver burden over time and qualitative studies about the daily hardships of family caregivers of stroke survivors. Given the scarcity of support programs in Brazil, greater knowledge of the emotional and social circumstances of family caregivers of stroke survivors is needed.

### Contributions to nursing

Nurses working in home care and in Primary Health Care services should assess social isolation and general strain among non-spouse caregivers. Knowledge of both such hardships is needed to better inform Brazilian public health policy and program developers about the need to expand formal and/or employment support services for this group. We also recommend that nurses make support group referrals during SCU-Stroke discharge planning to help non-spouse caregivers see that they are not alone in their struggles. Our findings pinpoint promising areas for anticipatory family care planning with respect to hospital-to-home transitions. Given the scarcity of support programs in Brazil, these are equally important nursing considerations.

## CONCLUSION

This is the first Brazilian study to assess perceived burden among spouse and non-spouse caregivers who are caring for older adults with stroke-induced-dependency over time. Non-spouse caregivers experienced significantly higher levels of isolation over time and General Strain 2 months after taking over a caregiver role. Despite already receiving support from outside sources, this group felt more isolated and strained with respect to a decreased social life, a lack of time for life pursuits and the self, and own-health deterioration.

Our over-time findings draw necessary attention to the hardships that middle-aged non-spouse caregivers face, many of whom are likely to be first-time caregivers. Caregivers of frail older people are more vulnerable to isolation and loneliness^(46-47)^. Ongoing assessments of caregiver burden among this group after a loved one is discharged from a SCU-Stroke are imperative. Higher levels of social support seem to help this group better recover from stress and psychological distress^(48)^. Nurses could provide non-spouse caregivers with everyday problem-solving support to help them secure stolen moments for own-health promotion. This may help non-spouse caregivers feel less ‘tied-down’ in the absence of formal support.
